# Bis-Homoleptic Metal Complexes of a Tridentate Ligand with a Central Anionic Sulfonamide Donor

**DOI:** 10.3390/molecules30163378

**Published:** 2025-08-14

**Authors:** Mathias L. Skavenborg, Christine J. McKenzie

**Affiliations:** Department of Physics, Chemistry and Pharmacy, University of Southern Denmark, 5230 Odense, Denmark; skavenborg@sdu.dk

**Keywords:** bis-homoleptic complexes, tridentate ligand, sulfonamide ligand, electrochemistry

## Abstract

Redox-active manganese, iron, and nickel complexes of pyridin-2-ylsulfonyl-quinolin-8-yl-amide (psq) provide information for assessing the electronic and structural properties of this new tridentate ligand. Single-crystal X-ray structures show that psq coordinates in a meridional mode with a trigonal geometry for the central deprotonated sulfonamide N donor. With the structures described here, there are now five structures known for hexacoordinated bis-homoleptic complexes of psq. All show the same geometry. No *fac* isomer, although feasible, has been structurally characterized. The geometrical parameters for [M(psq)_2_]^0/+^ are surprisingly close to those for archetypical [M(terpy)_2_]^2+/3+^ (terpy =2,2′:6′,2″-terpyridine) complexes, with octahedral distortion parameters indicating a geometry that is slightly closer to a regular octahedral. The Fe(II) complex, however, bucks this trend, consistent with the magnetic susceptibility measurements indicating a high-spin *S* = 5/2 state, which stands in contrast to low-spin [Fe(terpy)_2_]^2+^. This is rationalized by the *trans* secondary sulfonamide donors being weaker π acceptors compared to central terpy pyridine donors. An overall two-integer reduced charge for the complexes is consistent with the Co^II^/Co^I^, M^III^/M^II^ M = Mn, Fe, Co, and Mn^IV^/Mn^III^ redox events being ca. 600–900 mV more cathodic compared to the corresponding events for [M(terpy)_2_]^2+^.

## 1. Introduction

**Scheme 1:**
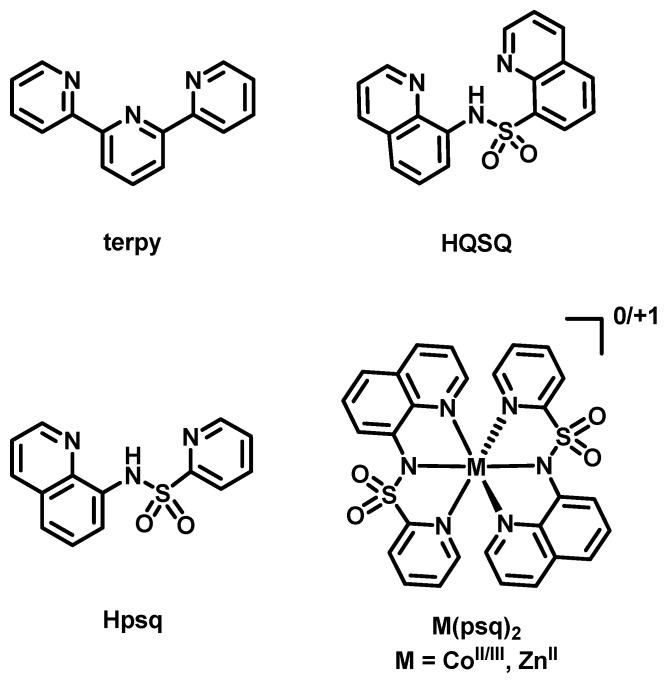
2,2′:6′,2″-terpyridine (terpy) [8]; the protonated proligands *N*-(quinolin-8-yl)quinolin-8-sulfonamide, (HQSQ) [7], and pyridin-2-ylsulfonyl-quinolin-8-yl-amide (Hpsq); and the known hexacoordinated bis-psq complexes [3].

Tridentate meridionally coordinating ligands, often called pincer ligands, are used in a wide range of applications, from catalysts to photoactive compounds, and significant effort has been devoted to tuning their electronic properties [1,2]. This includes using various electron-donating and -withdrawing substituents and donor atoms, e.g., NNN, PNP, (PO)C(OP), NCN, CNC, and SNS (N = *N*-heteroaromatics, aromatic and aliphatic amines, C = carbenes, P = phosphines, PO = phosphine oxides, and S = sulfur donors), in typically symmetric systems [2]. A new potentially tridentate scaffold containing a central anionic sulfonamide-N donor, pyridin-2-ylsulfonyl-quinolin-8-yl-amide (psq), was reported recently [3]. The protonated proligand Hpsq is shown in Scheme 1. In the handful of cobalt(II/III), zinc(II), and copper(I/II) complexes now characterized, the central sulfonamide group is deprotonated. The two chelate rings are different when psq is a tridentate ligand, and this lack of symmetry is a feature that could be exploited in the design of catalysts for asymmetric catalysis [4]. Interesting activities have been observed: a dimeric 1:1 Cu(I):psq complex ([Cu_2_(psq)_2_]) can activate O_2_ [5], and monomeric 1:1 Cu(II):psq complexes serve as pre-catalysts for electrocatalytic oxygen reduction [6]. The bis-homoleptic cobalt complex shows a remarkable span of 1.75 V between its reversible Co^III^/Co^II^ and Co^II^/Co^I^ couples [3]—an attractive property sought for energy storage in redox flow batteries.

Psq exhibits a meridional coordination geometry in the structures of the bis-homoleptic complexes [Co^II^(psq)_2_], [Co^III^(psq)_2_]^+^, and [Zn^II^(psq)_2_] (Scheme 1) and for one of the psq ligands in [Cu^II^(psq)_2_] (the other acts as a bidentate ligand with the pyridine donor uncoordinated to this Jahn–Teller metal ion) [6]. When tridentate, the coordination geometry is unexpectedly like that furnished by archetypical tridentate 2,2′:6′,2″-terpyridine (terpy, Scheme 1). In contrast to planar aromatic terpy, a facial geometry would seem to be equally feasible for psq, given that planarity is not electronically dictated since the backbone tetrahedral sulfone S atom is not conjugated with the aromatic rings. Indeed, this is the case for the two structurally characterized complexes of *N*-(quinolin-8-yl)quinolin-8-sulfonamide [7] (HQSQ, Scheme 1), which in our view can be regarded as the most closely related ligand to psq in the literature. Compared to psq, however, QSQ does have an additional C atom in one of the chelate rings, which may better accommodate facial geometries. Such a facial arrangement has not yet been observed for the structures of psq, which, with this contribution, now amount to seven, of which five, including the two we report here, are bis-homoleptic complexes. For this class of compounds, we count the potential for five *fac* diastereoisomers, three of which are chiral, and one *mer* diastereoisomer (SI, Scheme S2), hence 10 isomers altogether, making for a very “rich” (i.e., annoying) structural chemistry. Interestingly, the single-crystal X-ray structure of the protonated proligand suggests that it is actually pre-organized for *fac* coordination with the sulfonamide NH group already in a close to trigonal N geometry. The angle between the planes of the rings of terminating aromatic N donors is 95° [3]. It is feasible, however, that an intermolecular π-π stacking between quinoline groups of Hpsq was a driving force for this arrangement in the absence of metal coordination.

Terpy is a rigid, planar aromatic system whose complexes exhibit rich photophysical and electrochemical properties. It was first synthesized in 1932 by G. Morgan and F. H. Burstall [8] and has been ubiquitously used for the construction of bis-homoleptic complexes of d-block metal ions. Attesting to the significance of these complexes in the fields of coordination, supramolecular, and photochemistry are 294 crystal structures of bis-terpy complexes in the CCDC [9] and an additional 1143 structurally characterized derivatives in which one or more of the H atoms of any of the rings are substituted. A relatively wide range of oxidation states and geometries has been observed, from Ti^4+^ in [Ti(terpy)_2_]^2+^ [10] to Co^1+^ in [Co(terpy)_2_](PF_6_) [11]. [Fe(terpy)_2_]^2+^ is a low-spin *S* = 0 system at room temperature, but a spin state switch to high-spin *S* = 2 can be photoinduced [12,13], and the system is used in photocatalysis in the synthesis of carbazoles [14]. Ni(terpy)_2_ has applications in photocatalytic [15] and electrocatalytic [16] CO_2_ reduction. [Mn(terpy)_2_]^2+^ has been deployed as a precursor [17] for an O_2_-evolving catalyst [18], and para-substituted analogues have been tested as biomimetic analogues for superoxide dismutase [19]. Substitution of the periphery H-atoms impacts the redox potentials [19,20,21], electrochemical stability [22,23], electronic structure [24], and photochemistry [25] of the complexes. Given the extensive utility of terpy, the complexes of psq may be interesting for further exploration for many of these applications. The contrasting features, namely (i) a quinoline group with attendant ligand redox non-innocence, (ii) the lack of chemical symmetry around the central N donor, (iii) an overall charge two integers lower than those for the corresponding bis-terpy systems, (iv) second coordination sphere interactions involving the periphery sulfone group, and lastly (v) inherent chirality of the coordinated sulfonamide N atom, offer new directions for tuning physical properties and chemical reactivity for bis complexes compared to terpy systems. While the anionic nature of psq can be expected to lead to stronger L-M electrostatic interaction, the strongly electron-withdrawing effects of the sulfonyl (SO_2_) and the quinoline donor can be anticipated to counter this effect.

To further elucidate the psq system, we expanded the series of bis-psq complexes with Ni(II), Fe(II), and Mn(II) complexes. These have been structurally, spectroscopically, and electrochemically characterized to glean more information about their electronic structures.

## 2. Results and Discussion

### 2.1. Synthesis and Characterization

**Figure 1:**
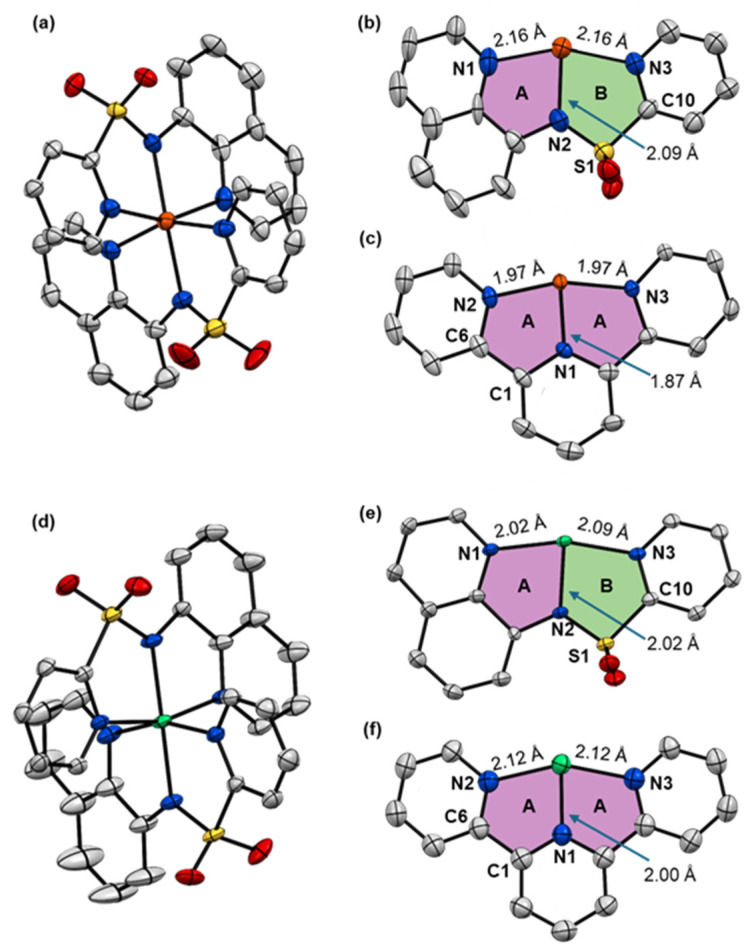
(**a**) Crystal structure of Fe(psq)_2_. View of one ligand in (**b**) Fe(psq)_2_ and (**c**) [Fe(terpy)_2_](ClO_4_)_2_ (CCDC ref code: CECCOG) [27]. (**d**) Crystal structure of Ni(psq)_2_(THF)_1.5_. View of one ligand in (**e**) Ni(psq)_2_(THF)_1.5_ and (**f**) [Ni(terpy)](ClO_4_)_2_(H_2_O) (CCDC ref code: IQUHAF) [28]. Thermal ellipsoids are shown at 50% probability. Hydrogen atoms, co-crystallized THF, water, MeCN, and ClO_4_^−^ molecules are omitted for clarity. Gray = carbon, blue = nitrogen, red = oxygen, yellow = sulfur, orange = iron, and light green = nickel. The purple (A) and green (B) areas indicate the different chalate rings observed in M(terpy)_2_ and M(psq)_2_

Green Ni(psq)_2_ and yellow Mn(psq)_2_ were prepared by the reaction of the metal acetates with two equivalents of Hpsq in an acetone/aqueous solution (50:50). The synthesis of red Fe(psq)_2_ was similar except that the presence of ascorbic acid was necessary for avoiding formation of the green [Fe(psq)_2_]^+^. Crystals of Fe(psq)_2_ were grown from a CH_2_Cl_2_/hexane solution, and the single-crystal X-ray structure is shown in Figure 1a. Coordination through the quinoline, pyridine, and sulfonamide nitrogen results in an irregular octahedral coordination sphere where the trans-orientated Fe-N_sulfonamide_ (2.09(1) Å) distances are shorter than the Fe-N_pyridine_ (2.18(1) Å) and Fe-N_quinoline_ (2.17(1) Å) distances. The average bond lengths (Fe-N) in Fe(psq)_2_ are 2.14 Å, similar to other hexacoordinated all N-donor high-spin Fe(II) complexes [26] and longer than the Fe-N bond lengths in the low-spin [Fe(terpy)_2_](ClO_4_)_2_ (1.87–1.97 Å). Ni(psq)_2_ was crystallized from slow evaporation of the crude product dissolved in tetrahydrofuran (THF) (Figure 1d) and exhibits the same trends in the solid state as Fe(psq)_2_, where the average Ni-N_quinoline_ (2.077Å) and Ni-N_pyrdine_ (2.101 Å) bonds are longer than the average Ni-N_sulfonamide_ (2.022 Å). As mentioned in the Introduction, both M(psq)_2_ were crystalized as *mer* isomers (SI, Scheme S2). The main differences between the solid-state structures of M(psq)_2_ and M(terpy)_2_ (M = Fe^2+^, Ni^2+^) are the five-membered chelate rings constituting the MNCCN (M = Fe^2+^ or Ni^2+^) rings in [Fe(terpy)_2_]^2+^ (ring A) and the MNCCN (ring A) and MNSCN (ring B) rings in M(psq)_2_. The average NCCN lengths in ring A is similar for M(psq)_2_ (4.3 ± 0.03 Å) and [M(terpy)_2_]^2+^ (4.2 ± 0.008 Å), whereas the average NS(O)_2_CN bond length in Ring B is 0.5 Å greater (4.7 ± 0.02 Å), consequently forming a larger chelate ring. Additionally, the N2-S1-C10 angle (∠ = 100°) is more acute than that of N1-C1-C6 (∠ = 111°) in the NCCN chelate rings found in both Fe(psq)_2_ and [Fe(terpy)_2_]^2+^. As expected, a longer S1-C10 (1.77 Å) distance is found in the sulfonamide compared to the 1.456 Å for the C1-C6 bond between the pyridine rings in [Fe(terpy)_2_]^2+^. In addition, the N1-Fe-N3 angle is more acute in Fe(psq)_2_ (∠ = 155°) compared to the N2-Fe-N3 angle in [Fe(terpy)_2_]^2+^ (∠ = 162°). These factors influence the difference in the spin state. The pyridyl and quinoline rings are close to co-planar (∠_py-qu_ = 12.6°) but slightly more out of plane compared to M(psq)_x_ (M = Zn^2+^ (x = 2), Co^2+/3+^ (x = 2), and Cu^2+^ (x = 1)), which is ∠_py-qu_ = 6°–9.5° (see SI Tables S1–S5 for crystallographic details and selected bond angle/length in the supporting information). The elemental analysis for Mn(psq)_2_ is consistent with a 1:2 metal–ligand complex, and its infrared spectrum is similar to those for M(psq)_2_ (M = Ni, Fe, SI, Figure S1). Thus, we propose the same *mer* coordination geometry in the solid state as that found for its Co, Zn, Ni, and Fe analogues. We were, however, unable to obtain crystals suitable for single-crystal X-ray diffraction.

**Figure 2:**
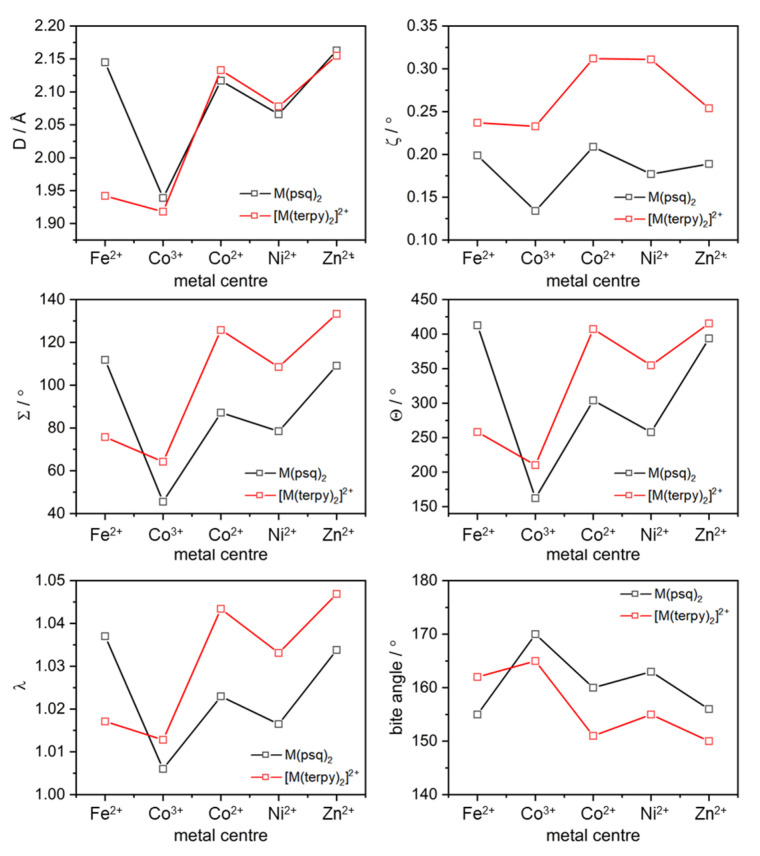
The octahedral distortion parameters (D, ζ, ∑, and θ). Bite angles and Robinson’s quadratic elongation parameter (λ) as a function of metal center in M(psq)_2_ and [M(terpy)_2_]^2+^.

Octahedral distortion parameters (D, ζ, ∑, and θ) [29,30,31] calculated using OctaDist [32] and Robinson’s quadratic elongation parameter (λ) [33], which describes how much a hexacoordinated metal complex deviates from the perfect octahedral geometry, are visualized in Figure 2 (SI, Table S5 for data). The distortion parameters for M(psq)_2_ (Co^III/II^, Fe^II^, Ni^II^, and Zn^II^) follow the same trends as the analogous [M(terpy)_2_]^2+^ complexes [27,28,34,35,36], with most of the values being lower, indicating a marginally closer to ideal octahedral geometry for the M(psq)_2_ complexes. However, Fe(psq)_2_ is an outlier with respect to all the parameters due to longer mean bond lengths and a more distorted octahedral geometry compared to [Fe(terpy)_2_]^2+^.

### 2.2. Magnetic Susceptibility

**Figure 3:**
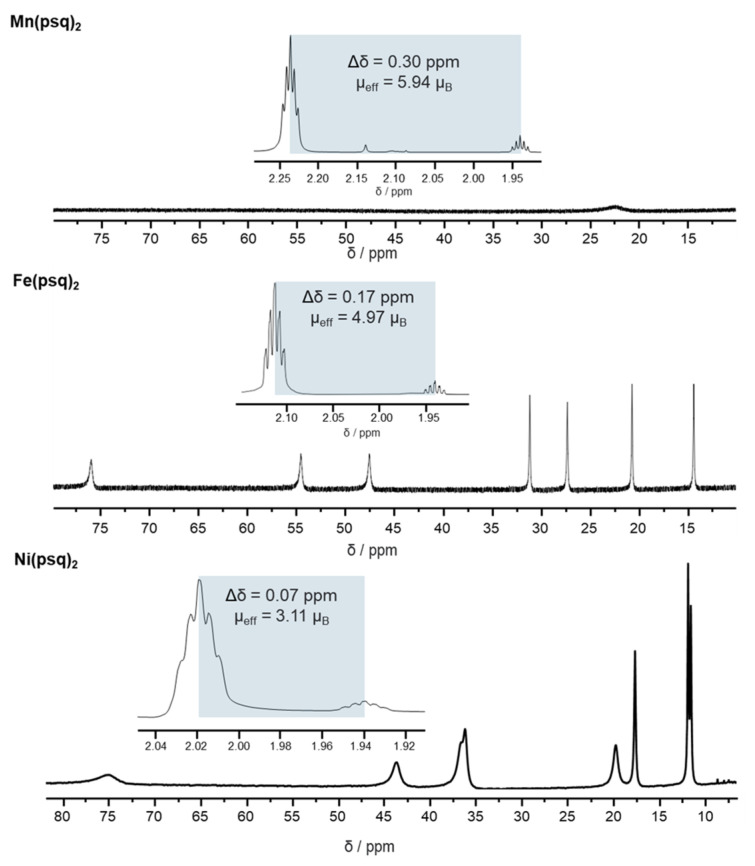
^1^H-NMR spectra of Mn(psq)_2_, Fe(psq)_2_, and Ni(psq)_2_ in CD_3_CN in the region 10–80 ppm with an inset of the CHD_2_CN signals in the presence and absence of paramagnetic species.

The ^1^H-NMR spectra (Figure 3) of Fe(psq)_2_, Ni(psq)_2_, and Mn(psq)_2_ recorded in CD_3_CN reveal paramagnetically shifted and broadened signals in the range 100 to −100 ppm, as can be expected. There are seven signals corresponding to chemically inequivalent H atoms in Fe(psq)_2_ (76, 54, 47, 31, 27, 21, and 14 ppm) and eight (75, 44, 36.6, 36.2, 19, 18, 11.9, and 11.6 ppm) in the spectrum of Ni(psq)_2_. Only one broad singlet is observed at 22.5 ppm in the spectrum of Mn(psq)_2_. By comparison, the ^1^H NMR spectra of the structurally equivalent diamagnetic Zn(psq)_2_ and [Co(psq)_2_]PF_6_ analogues [3] show 10 resolved signals in the aromatic region (7.5–8.8 ppm) for the 10 chemically distinct H atoms. Furthermore, distinct second-order couplings are discernible. Thus, not only are some signals apparently so broad that they are not observed for the paramagnetic bis-psq Fe(II), Ni(II), and Mn(II) complexes, but all second-order couplings are also completely unresolved. The magnetic susceptibility for all three complexes was measured using the Evans method [37], where the chemical shifts of residual CHD_2_CN were used as a diamagnetic reference. The chemical shifts (Δδ) of 0.07 ppm for Ni(psq)_2_, 0.17 ppm for Fe(psq)_2_, and 0.30 ppm for Mn(psq)_2_ in CD_3_CN are consistent with effective magnetic moments of 3.1, 5.0, and 5.9 µ_B_ and high-spin Ni^II^ (*S* = 1), Fe(psq)_2_ (*S* = 2), and Mn^II^ (*S* = 5/2) systems. Measurements for Fe(psq)_2_ were also performed in CDCl_3_ and DMSO-*d*_6_ (SI, Figure S2) and indicate that the high-spin (*S* = 2) state is solvent-independent.

### 2.3. Electrochemistry

**Figure 4:**
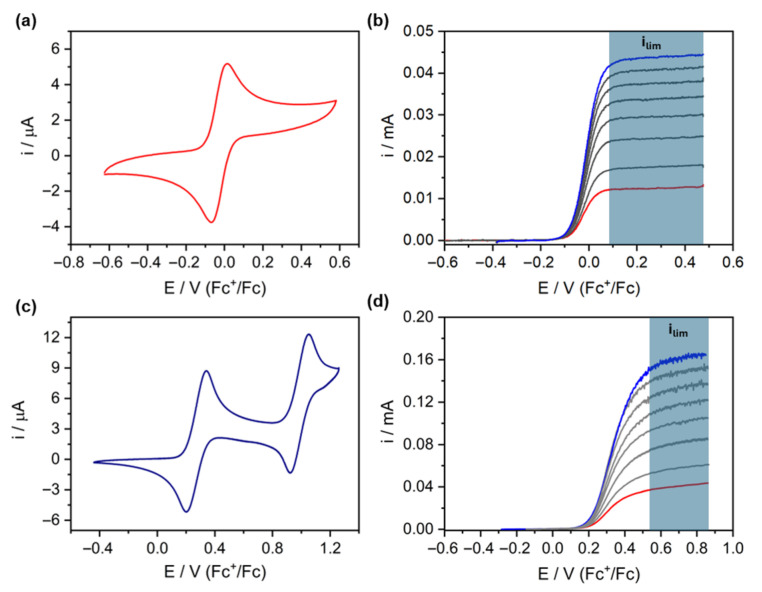
Cyclic voltammograms in MeCN (0.1 M TBAPF_6_, glassy carbon electrode) of (**a**) Fe(psq)_2_ and (**c**) Mn(psq)_2_. LSV-RDE voltammograms of (**b**) Fe(psq)_2_ and (**d**) Mn(psq)_2_. All potentials a reported vs. the ferrocene/ferrocenium couple.

**Figure 5:**
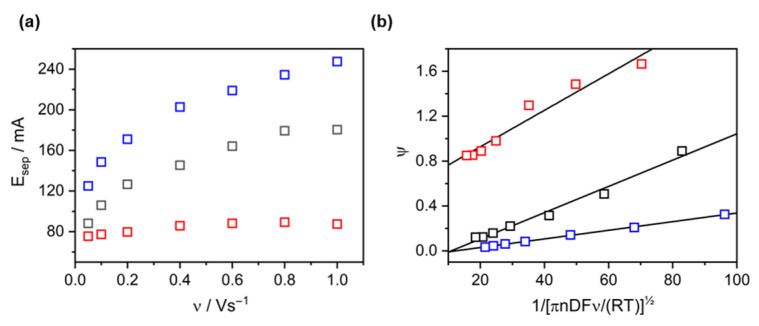
(**a**) Peak separation as a function of scan-rate and (**b**) Ψ as a function of 1/[πnDFν/(RT)]^½^ for Fe^II^ (red), Mn^III^ (blue), and Mn^IV^ (black).

Cyclic voltammograms (CV) for Fe(psq)_2_ and Mn(psq)_2_ vs. ferrocene/ferrocinium (Fc^+^/Fc) recorded in acetonitrile are shown in Figure 4. The oxidation at −0.009 V and reduction at −0.069 V indicate a Fe^III^/Fe^II^ redox potential for Fe(psq)_2_ very close to the Fc^+^/Fc potential (*E_½_* −0.03 V vs. Fc^+^/Fc). This redox event ranges from −0.0036 to 0.039 V vs. Fc^+^/Fc depending on the solvent medium (dichloromethane, dimethylformamide, dimethyl sulfoxide, acetonitrile, and tetrahydrofuran; see SI Figure S3). Cyclic voltammograms recorded at different scan rates (SI, Figure S4 and Figure 5) reveal small increases in the separation of the peak-to-peak potentials (E_sep_ = E_pa_ − E_pc_) from 75 to 89 mV. Thus, the electron transfer is quasi-reversible, and in fact, the peak separation is relatively small compared to many if not most high-spin Fe^III^/Fe^II^ systems, for which quasi-irreversibility is common due to a higher propensity for EC processes compared to low-spin complexes like ferrocene and iron hexacyanides. These show exemplary Fe^III^/Fe^II^ reversibility—a property that has found numerous electrochemical applications (electrochemical reference [38], sensors [39,40], redox mediators [41], and charge storage [42]). We speculate that Fe(psq)_2_ and its potentially relatively synthetically accessible derivatives (through substitutions on the pyridine and quinoline rings) could find ferrocene-like electrochemical applications but with the additional property of paramagnetism in both redox states.

The CV of Mn(psq)_2_ shows redox events at 0.25 V and 0.99 V vs. Fc^+^/Fc assigned to the Mn^III^/Mn^II^ and Mn^IV^/Mn^III^ couples. Zn(psq)_2_ is redox silent in this window [3], supporting manganese-centered processes. The linear sweep voltammogram at a rotating disk electrode (SI, Figure S5) confirms that one electron is involved in each event. Peak-to-peak separations of 149 mV (Mn^III^/Mn^II^) and 105 mV (Mn^IV^/Mn^III^) indicate quasi-reversible redox processes. Diffusion coefficients of 6.72 × 10^−6^ cm^2^/s and 9.06 × 10^−6^ cm^2^/s for Fe(psq)_2_ and Mn(psq)_2_, respectively, which were determined using the diffusion limited current (i_lim_, Figure 4) from rotating disk electrode (RDE) voltammetry experiments [43], are in good agreement with the values of 7.12 × 10^−6^ cm^2^/s and 7.95 × 10^−6^ cm^2^/s obtained using chronoamperometry [44] (SI, Figures S6 and S7). The diffusion coefficient determined using chronoamperometry of the charged M(III) species [Fe(psq)_2_]^+^ and [Mn^III^(psq)_2_]^+^ are 7.06 × 10^−6^ cm^2^/s and 9.85 × 10^−6^ cm^2^/s, respectively, and very close to the observed values for the M^II^ oxidation states. For [Mn^IV^(psq)]^2+^, a lower diffusion coefficient was determined at 2.39 × 10^−6^ cm^2^/s (SI, Figure S7). A larger solvation sphere might be expected with the higher charge and presumably slightly smaller cation, and this could be expected to slow diffusion. The CV of Ni(psq)_2_ showed an irreversible Ni^III^/Ni^II^ process (SI, Figure S8) with an oxidation at 0.44 V and a reduction at 0.30 V vs. Fc^+^/Fc with i_pa_/i_pc_ = 5.7.

The E_sep_ values as a function of scan rate were in the range of 125–247 mV and 96–192 mV for the Mn^III^/Mn^II^ and Mn^IV^/Mn^III^ couple, respectively (Figure 5). The standard heterogeneous electron transfer rate constant (k_0_) was determined from Nicholson–Shain analysis [45] by relating the dimensionless parameter (ψ) with k_0_ (Equation (1)).

(1) $$k_{0}=\psi {\left[\frac{\pi D_{o}\mathrm{nFv}}{\mathrm{RT}}\right]}^{½}$$

ψ was determined using the separation between the peak potentials [46]. The calculated k_0_ was 0.016 cm/s for Fe(psq)_2_. This is lower than [Fe^III^(X-sal)_2_trien]^+^ (X = 5-OCH_3_, 3-OCH_3_, H, 3-NO_2_ and 5-NO_2_), which are between 0.024 and 0.047 cm/s in isobutyl nitrile [47]. The k_0_ for the manganese Mn^III^/Mn^II^ was lowest at 0.0026 cm/s, indicating the slowest reaction between the complex and the electrode surface. This is likely due to the reorganization needed for the conversion between the d^5^ (Mn^II^) and the Jahn–Teller distorted d^4^ (Mn^III^) species. The conversion between the Mn(III) and Mn(IV)species was slightly faster (0.0056 cm/s).

**Figure 6:**
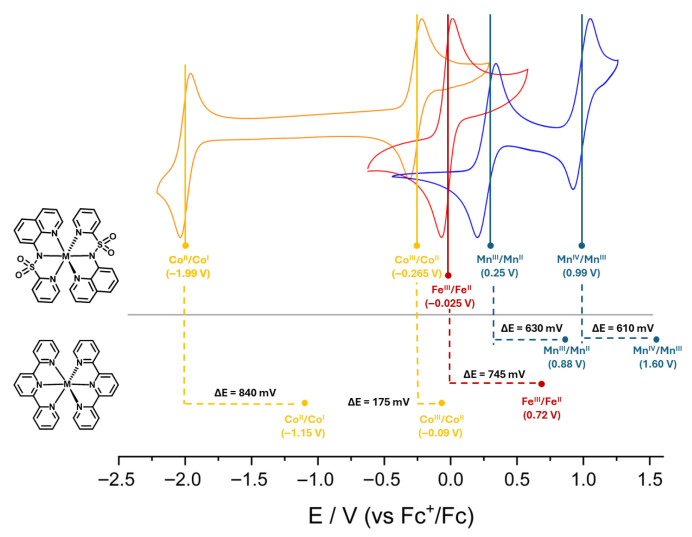
Measured metal-based redox potentials of M(psq)_2_ (Co = orange, Fe = red and Mn = blue) and reported metal potentials of [M(terpy)_2_]^2+^ in acetonitrile.

The metal-centered redox events for M(psq)_2_ (Co, Fe, Mn) span a whopping 2.98 V in acetonitrile. Some of the CVs are shown in Figure 6, along with the corresponding values for [M(terpy)_2_]^2+^ (Co, Fe, Mn), which span 2.75 V [48,49,50]. Psq stabilized the higher oxidation states (Mn^III^, Mn^IV^) by 610 and 630 mV, respectively, whereas the lower oxidation states (Co^I^) were destabilized by 840 mV. While the overall charge of the complexes influenced the values of the redox potentials, the central axial donors in psq and terpy were significantly different. Given the negative charge, the sulfonamide N can be expected to be a stronger σ donor compared to the central pyridine of terpy. In addition, this negative charge and resonance with the sulfonyl group will limit π backbonding, presumably making the sulfonamide a weaker π acceptor. This is clearly also reflected by the contrasting low- and high-spin states, respectively, of the corresponding bis-terpy and bis-psq complexes of Fe(II).

### 2.4. UV/Vis Spectrophotometry

**Figure 7:**
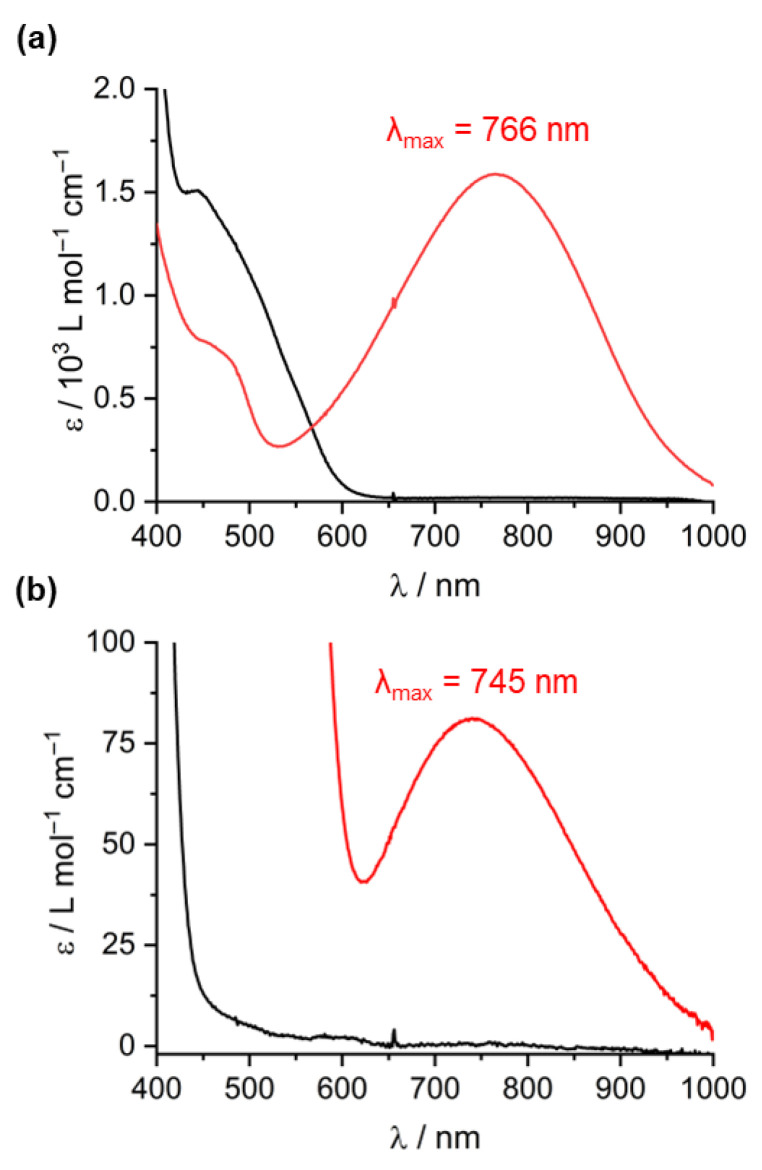
UV/Vis absorption spectra of (**a**) Fe(psq)_2_ (0.501 mM, black) and (**b**) Mn(psq)_2_ (2.12 mM, black). The red spectra are bis-psq Fe(III) and Mn(III) complexes observed after the reaction with one eq. CAN.

The UV/Vis absorbance spectra of Fe(psq)_2_ and Mn(psq)_2_ in MeCN are shown in Figure 7. An absorbance at λ_max_ = 367 (ε_0_ = 4.1 × 10^3^ M^−1^ cm^−1^) with a shoulder at λ_max_ = 466 (ε_0_ = 1.5 × 10^3^ L mol^−1^ cm^−1^) in the 200–600 nm region (SI, Figure S9) is found in the spectrum of Fe(psq)_2_. This region is less rich in features compared to the spectrum of [Fe(terpy)_2_](BF_4_) [51] in MeCN. When one equivalent of the one-electron oxidant, ceric ammonium nitrate (CAN), was added to this solution, stoichiometric oxidation to the green [Fe(psq)_2_]^+^ occurred, as evidenced by a new LMCT band at λ_max_ = 766 nm (ε_0_ = 1.5 × 10^3^ M^−1^ cm^−1^). Only limited structural and electronic identification of [Fe(terpy)_2_]^3+^ exists [52,53]; however, the spectrum of a light green [Fe(terpy)_2_](ClO_4_)_3_ in conc. H_2_SO_4_ has an absorption at λ_max_ = 702 nm (ε_0_ = 740 cm^2^ mol^−1^) [54]. The yellow Mn^2+^ is also featureless between 500 and 1000 nm, with a sole absorption at 370 nm (ε_0_ = 9.2 × 10^3^ M^−1^ cm^−1^) (SI, Figure S9). Three new absorption bands appear at 405 nm (ε_0_ = 5.1 × 10^3^ M^−1^ cm^−1^), 511 nm (ε_0_ = 0.9 × 10^3^ M^−1^ cm^−1^), and 745 nm (81 L mol^−1^ cm^−1^) upon the addition of CAN (Figure 6, SI Figure S9c). These new bands are assigned to the d-d transitions in the resultant Mn^3+^ species, and these are blue-shifted compared to the d-d bands for [Mn(terpy)_2_]^3+^ (λ_max_ = 455 nm, 360 nm, and 320 nm) [48]. The spectrum does not change upon the addition of a second equivalent of CAN, consistent with CAN not being a strong enough oxidant (E_red_ = 1.61 V vs. NHE) [55,56] to form a [Mn(psq)_2_]^2+^ species (Mn^IV^/Mn^III^ for Mn(psq)_2_ is E_½_ = 1.63 vs. NHE (see conversion in the Methods and Materials section)). Ozone (E_red_ = 2.07 V vs. NHE) is necessary to oxidize [Mn(terpy)_2_]^2+^ to Mn^IV^ with oxo-bridged clusters identified [57,58] and is theoretically also a strong enough oxidant for Mn^III^(psq)_2_. The absorbance spectrum of Ni(psq)_2_ is typical for octahedral Ni(II) complexes with three absorbances [λ_max_, nm (ε_0_, M^−1^ cm^−1^): 375 (5.2 1.5 × 10^3^), 550 (30.6), 911 (40.1) SI, Figure S9a].

## 3. Methods and Materials

### 3.1. General

All chemicals were used as received from the vendors. Hpsq was prepared according to the literature procedure [3]. Unless otherwise stated, ^1^H- and ^13^C{H}-NMR samples were prepared in deuterated solvents, and the spectra were recorded on a Jeol JNM-ECZR 500 MHz spectrometer (Akishima, Japan) at ambient temperature; the data were processed with MestReNova version 12.01-20560. ESI-MS spectra were recorded on a nano spray Bruker microOTOFQ II (Billerica, MA, USA) in positive ionization mode, and mMass version 5.5.0 was used for visualization of the obtained data. IR spectra were recorded on an Agilent Cary 603 FTIR (Santa Clara, CA, USA). OriginPro version 2020b was used for general data analysis and visualization. Elemental analysis was measured using a FlashEA 1112 NC Analyzer (Thermo Scientific, Waltham, MA, USA) at Copenhagen University. Crystals used for single-crystal X-ray diffraction were taken directly from the mother liquor and coated in Fomblin^®^Y (Sigma-Aldrich, St. Louis, MO, USA) or Paratone oil (Sigma-Aldrich) to allow the crystal to adhere to the mounting loop. X-ray crystal diffraction data were collected at 100(1) K on a Synergy, Dualflex, AtlasS2 diffractometer (Rigaku, Tokyo, Japan) using CuK_α_ radiation (*λ* = 1.54184 Å) and the CrysAlis PRO 1.171.42.90a suite and corrected for Lorentz polarization effects and absorption. Using shelXle [59] and Olex2 [60], all the structures were solved by dual-space methods (SHELXT [61]) and refined on *F*^2^ using all the reflections (SHELXL-2019/2 [62]). All the non-hydrogen atoms were refined using anisotropic atomic displacement parameters; hydrogen atoms bonded to carbon were inserted at calculated positions using a riding model. Crystallographic parameters for all the complexes, along with any additional refinement details, are described in the ESI. Electrochemistry was recorded using a Biologic S300 potentiostat (Biologic, Seyssinet-Pariset, France). Cyclic voltammetry (CV) was conducted in degassed (N_2_) solutions of acetonitrile with tetrabutylammonium hexafluorophosphate as the supporting electrode using a standard three-electrode setup with a glassy carbon working electrode (GCE, Ø = 3 mm), Pt-wire counter electrode, and an Ag/AgCl as a pseudo reference electrode. A CV was recorded of ferrocene before and after each section of the experiments and used to calculate the reported potential. The Ag/AgCl reference electrode was converted to NHE using the following equation:

(2) $$E_{\mathrm{NHE}}\;=\;E_{\mathrm{Ag}/\mathrm{AgCl}\;}-\;0.205\;V$$

Nicholson–Shain analysis was conducted using the same setup as described above, and the peak separations used to calculate k_0_ were corrected for solution resistance using impedance spectroscopy by finding the real axis in a Nyquist plot. Cotrell plots were constructed using Equation (3) below. A potential that was 300 mV higher or lower than the redox event was used to fully oxidize or reduce the complex in question.

(3) $$i=\frac{\mathrm{nFAC}\sqrt{D}}{\sqrt{\pi t}}$$

The rotating disk experiments were conducted using a GCE (Ø = 3 mm), and the diffusion coefficient was determined using the Koutecký–Levich equation shown below. The diffusion coefficients from the Cotrell plots were used to determine the number of electron(s) involved in the process.

(4) $$\frac{1}{i_{\lim}}=\frac{1}{i_{k}}+\left(\frac{1}{0.62\mathrm{nFA}D^{\frac{2}{3}}\upsilon^{-\frac{1}{6}}C}\right)\omega^{-\frac{1}{2}}$$

Magnetic susceptibilities were measured in the given solvent (DMSO-*d*_6_, CD_3_CN, or CDCl_3_) using the Evans method [37]. A quartz NMR tube was charged with solutions of the complexes and a capillary tube containing pure solvent. The mass susceptibility (Equation (5), where χ_g_ in cm^3^ g^−^^1^) was calculated using the shifts in NMR signals for the solvent containing the paramagnetic analyte and that in the capillary tube.

(5) $$\chi_{g}=\frac{3∆\delta /10^{6}\;}{4\pi c(1+{\;\delta }_{\mathrm{ref}}/10^{6})}$$

Equation (5) is used for dilute solutions [63,64]. Multiplying χ_g_ with the molecular weight (MW) yields the molar susceptibility χ_M_ (cm^3^ mol^−^^1^), which was corrected for the diamagnetic contributions by subtracting χ_M,dia_ (= −0.5 × MW × 10^−^^6^ cm^3^ mol^−^^1^) [65].

### 3.2. Synthesis

**Fe(psq)_2_:** FeCl_2_ (0.040 g, 316 µmol) in water was added dropwise to an acetone solution of Hpsq (0.200 g, 700 µmol), and the resulting dark solution was stirred for 10 min. A red precipitate was isolated after the addition of excess ascorbic acid (0.166 g, 945 µmol) dissolved in H_2_O (3 mL). The precipitate was isolated and rinsed with water (3 × 15 mL) and ether (3 × 15 mL); yield (80%, 0.175 g, 280 µmol). Single crystals for X-ray crystallography were obtained by recrystallization from hexane–DCM (1:1) (CCDC: 2474136). ^1^H-NMR (500 MHz, CD_3_CN) *δ* (ppm) = 76.0 (s, 1H), 54.5 (s, 1H), 47.5 (s, 1H), 31.1 (s, 1H), 27.3 (s, 1H), 20.6 (s, 1H), 14.3 (s, 1H), ESI-MS (pos. mode, MeCN) *m*/*z* = 625.0159 (625.0410, 17%, [Fe(psq)_2_]^+^), 646.9999 (647.0229, 100%, [Fe(psq)_2_+Na]^+^) 662.9737 (662.9968, 14%, [Fe(psq)_2_+K]^+^), 963.9843 (964.0175, 16%, [Fe_2_(psq)_3_]^+^), 1271.0081 (1271.0566, 23%, [Fe_2_(psq)_4_+Na]^+^), IR (FT-ATR diamond anvil) cm^−1^ = 1320, 1119 (S=O, str, s). Anal. calcd. (%) for C_28_H_20_N_6_FeS_2_O_4_: C: 53.61 H: 3.21 N: 13.40 S: 9.36. Found C: 53.00 H: 3.08 N: 12.92 S: 9.81.

**Mn(psq)_2_:** Hpsq (0.289 g, 1.04 mmol) was dissolved in acetone (5 mL) before Mn(OAc)_2_·4H_2_O (0.122 g, 0.490 mmol) in H_2_O (5 mL) was added to the solution. The resulting solid was isolated and rinsed with H_2_O (2 × 15 mL) and ether (2 × 15 mL) and isolated as the title compound (yield 90%, 0.275 g, 0.44 mmol). ESI-MS (pos. mode, MeCN): *m*/*z* 961.9877 (100%, [Mn_2_(psq)_3_]^+^ calcd 962.0237), 624.0217 (48%, [Mn(psq)_2_+H]^+^ calcd 624.0441). IR (cm^−1^): 1298, 1110 (S=O, str, s). Anal. calcd. (%) for C_28_H_20_N_6_NiS_2_O_4_: C: 53.61 H: 3.21 N: 13.40 S: 9.36. Found C: 53.41 H: 2.99 N: 12.98 S: 9.95.

**Ni(psq)_2_:** Hpsq (0.198 g, 0.694 mmol) was dissolved in acetone (5 mL) before Ni(OAc)_2_·4H_2_O (0.087 g, 0.350 mmol) in H_2_O (5 mL) was added to the solution. The resulting solid was isolated and rinsed with H_2_O (2 × 15 mL) and ether (2 × 15 mL) and isolated as the title compound. Recrystallization in THF afforded crystals of Ni(psq)_2_ suitable for X-ray crystallography (CCDC: 2474462) (yield 83%, 0.180 g, 0.287 mmol). ^1^H-NMR (500 MHz, CD_3_CN) *δ* (ppm) = 75.8 (bs, 1 H), 43.8 (s, 1H), 36.6 (s, 1H), 36.2 (s, 1H), 19.7 (s, 1H), 17.6 (s, 1H), 11.9 (s, 1H), 11.6 (s, 1H). ESI-MS (pos. mode, MeCN): *m*/*z* 1277.0088 (21%, [Ni(psq)_2_+2H+Na]^+^ calcd 1277.0731), 969.9844 (3%, [Ni(psq)_3_+2H]^+^ calcd 670.0339), 649.0009 (100%, [Ni(psq)_2_+Na]^+^ calcd 649.0233). IR (FT-ATR diamond anvil) cm^−1^ = 1329, 1115 (S=O, str, s). Anal. calcd. (%) for C_28_H_20_N_6_NiS_2_O_4_: C: 53.61 H: 3.21 N: 13.40 S: 9.36. Found C: 53.73 H: 3.19 N: 13.29 S: 9.78.

## 4. Conclusions

Psq represents a new class of chemically unsymmetrical tridentate ligand scaffold for which the coordination chemistry is unexplored. The structural, spectroscopic, and electronic properties of the bis-homoleptic complexes of several redox-active first-row transition metal ions using this ligand were investigated here. A strong preference for the ligands in M(psq)_2_ to adopt *mer* coordination remains unclear to us. With several structures showing this, it does seem too coincidental to be due to steric interactions in the solid state; however, we cannot rule out the fact that fac systems may be accessible in solution. In terms of bite angles, solid-state structures show that these complexes are unexpectedly geometrically like the ubiquitous bis-terpy complexes, with structural distortion parameters only slightly closer to an ideal octahedral geometry. Fe(psq)_2_ breaks this trend because it is a high-spin system (*S* = 2) in contrast with the [Fe(terpy)_2_]^+^ complex. The planes of the terminal aromatic rings deviate by 6–12° in the series of M(psq)_2_ structures. It is noteworthy that psq stabilizes a range of oxidation states that are 2.98 V apart, which increases the redox-potential window by 230 mV compared to the terpy complexes. The high-oxidation states in redox-active M(psq)_2_ are stabilized by ca. 600–700 mV compared with their [M(terpy)_2_]^+^ counterparts (M = Fe and Mn). This feature can be used to reduce the overpotentials of redox reactions involving Fe^III^, Mn^III^, or a Mn^IV^ species as redox catalysts, as we have shown for the Cu complexes. Interestingly, Fe(psq)_2_ shows a Fe^III^/Fe^II^ couple at a potential identical to that for the Fc/Fc^+^ couple, offering perspectives for application as an iron-based paramagnetic electrochemical reference and redox mediator. The system is amenable to incorporating supramolecular structures and electronic tuning through the facile modification of either or both the pyridine and quinoline donors. Finally, the quinoline group is potentially redox non-innocent, and since the system contains no proximal aliphatic C-H bonds, it may be robust for application in photochemical processes.

## Data Availability

The original contributions presented in this study are included in the article and Supplementary Material. Further inquiries can be directed to the corresponding author.

## Supplementary Materials

The following supporting information can be downloaded at: https://www.mdpi.com/article/10.3390/molecules30163378/s1, Figure S1. IR-spectra of Ni(psq)_2_ (green), Mn(psq)_2_ (blue), Fe(psq)_2_ (red) and Hpsq(black); Figure S2. ^1^H-NMR (80-10 ppm) of Fe(psq)_2_ in CDCl_3_ and DMSO-*d*_6_; Figure S3. Scan rate dependent CV of Fe(psq)_2_ (5 mM) in DCM, DMF, DMSO, MeCN, and THF (0.1 M TBAPF_6_) using a glassy carbon electrode; Figure S4. Cyclic voltammograms in MeCN (0.1 M TBAPF_6_) of Fe(psq)_2_ (a), Mn(psq)_2_ (c,d), and the corresponding Randles–Sevik plots for Fe(psq)_2_ (b) and Mn(psq)_2_ (d,f); Figure S5 Linear sweep voltammetry-rotating disk electrode (LSV-RDE) measurement for Mn(psq)_2_ in MeCN; Figure S6. Levich plot of Fe(psq)_2_ and Mn(psq)_2_ in MeCN (0.1 M TBAPF_6_) at applied potentials V; Figure S7. Cotrell plots for (a) Fe(psq)_2_, (b) Mn^III^/Mn^II^, and (c) Mn^IV^/Mn^II^ for Mn(psq)_2_; Figure S8. Cyclic voltammogram of Ni(psq)_2_ (5 mM) in MeCN (0.1 M TBAPF_6_) at a glassy carbon electrode with 100 mV/s; Figure S9. UV/Vis absorption spectra of (a) Ni(psq)_2_, (b) Fe(psq)_2_ in the presence (red) and absence (black) of cerium ammonium nitrate (CAN) and (c) Mn(psq)_2_ in the presence (red) and absence (black) of CAN; Scheme S1. Atom numbering of M(psq)_2_; Scheme S2. Facial and meridional isomers of M(psq)_2_ complexes. Three of the fac isomers (3, 4 and 5) have enantiomers (3’, 4’ and 5’). The mer isomers are *enantiomers* which are disordered in the crystal structures of the Co(II) and Co(III) complexes (CCDC ref codes SEFSUV, SEFSOP); Table S1. Crystal data and details of X-Ray diffractions for Fe(psq)_2_·0.33 CH_2_Cl_2_ and Ni(psq)_2_·1.5THF; Table S2. Selected bond distances measured for Fe(psq)_2_·0.33 CH_2_Cl_2_ and [Ni(psq)_2_]·1.5 THF; Table S3. *Cis* bond angles measured for Fe(psq)_2_·0.33CH_2_Cl_2_ and [Ni(psq)_2_]·1.5THF; Table S4. Trans bond angles measured for Fe(psq)_2_·0.33CH_2_Cl_2_, [Ni(psq)_2_]·1.5THF, Table S5. Octahedral distortion parameters (D, ζ, Σ, and θ) calculated using Octadist.

## Abbreviations

The following abbreviations are used in this manuscript:

CAN	Ceric ammonium nitrate
CCDC	Cambridge Crystallographic Data Centre
CV	Cyclic voltammetry
DCM	Dichloromethane
Fc	Ferrocene
HQSQ	*N*-(quinolin-8-yl)quinolin-8-sulfonamide
MeCN	Acetonitrile
Mer	Meridional
fac	Facial
SI	Supporting Information
Psq	Pyridin-2-ylsulfonyl-quinolin-8-yl-amide
TBAPF6	Tetrabutylammonium hexafluorophosphate
terpy	2,2′:6′,2″-Terpyridine
THF	Tetrahydrofuran
