# Supplementary material for: Bis-Homoleptic Metal Complexes of a Tridentate Ligand with a Central Anionic Sulfonamide Donor
Source: Molecules. 2025 Aug 14;30(16):3378. doi: 10.3390/molecules30163378 (PMC12388488; doi:10.3390/molecules30163378)
Supplement: Supplementary file 1 [file molecules-30-03378-s001.zip › molecules-3804693-supplementary.pdf]

Supporting Information

Bis-homoleptic Metal Complexes of a Tridentate ligand with a Central Anionic Sulfonamide Donor

Mathias L. Skavenborg,[a] *Christine J. McKenzie*[a,*]

[a] Department of Physics, Chemistry and Pharmacy, University of Southern Denmark, 5230 Odense M, Denmark, **E-mail:** mckenzie@sdu.dk.

Table S1. Crystal data and details of X-Ray diffractions for Fe(psq)2·0.33CH_2Cl_2 and $Ni(psq)_2$·1.5THF.

	$Fe(psq)_2\cdot0.33CH_2Cl_2$[a]	$Ni(psq)_2\cdot1.5THF$
Empirical formula	$C_{28.33}H_{19.66}FeN_6O_4S_2Cl_{0.66}$	$C_{36}H_{35}N_6NiO_6S_2$
Formula weight	623.46	770.53
Temperature/K	100.00(10)	100.01(10)
Crystal system	orthorhombic	monoclinic
Space group	$P2_12_12_1$	$P2_1/c$
a/Å	8.9133(5)	15.39520(10)
b/Å	13.8024(11)	21.8808(2)
c/Å	24.0592(17)	10.52310(10)
α/°	90	90
β/°	90	101.5690(10)
γ/°	90	90
Volume/Å3	2959.9(4)	3472.79(5)
Z	4	4
ρ_{calc} g/cm3	1.399	1.474
μ/mm-1	5.774	2.398
F(000)	1276.0	1604.0
Crystal size/mm3	0.135 × 0.034 × 0.019	0.14 × 0.035 × 0.026
Radiation	Cu Kα (λ = 1.54184)	Cu Kα (λ = 1.54184)
2Θ range for data collection/°	7.348 to 149.69	5.86 to 149.354
Index ranges	-11 ≤ h ≤ 11	-19 ≤ h ≤ 18
	-15 ≤ k ≤ 16	-27 ≤ k ≤ 27
	-30 ≤ l ≤ 24	-13 ≤ l ≤ 13
Reflections collected	22019	97777
Independent refl. [R_{int}, R_{sigma}]	5900 [0.0660, 0.0539]	7074 [0.0277, 0.0105]
Data/restraints/parameters	5900/649/371	7074/975/523
Goodness-of-fit on F2	1.068	1.043
Final R indexes [I>=2σ (I)]	R1 = 0.0913, wR2 = 0.2018	R1 = 0.0584, wR2 = 0.1633
Final R indexes [all data]	R1 = 0.1004, wR2 = 0.2075	R1 = 0.0615, wR2 = 0.1662
Largest diff. peak/hole / e Å-3	0.60/-0.56	1.02/-1.01
Flack Parameter	*0.041(5)*	-

[a] The diffraction data obtained for $Fe(psq)_2\cdot0.33CH_2Cl_2$ is of relatively poor quality. All attempts to obtain a crystal with better diffracting properties, e.g., by recrystallisation from other solvents, failed.

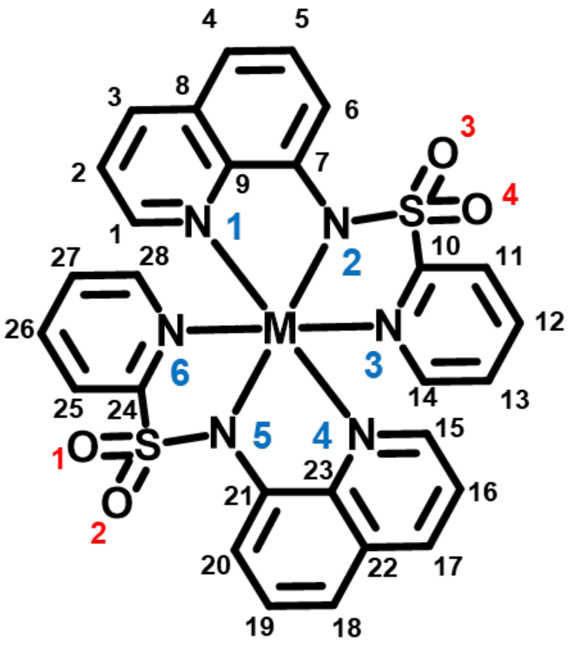

Scheme S1. Atom numbering of $M(psq)_2$

Table S2. Selected bond distances measured for $Fe(psq)_2 \cdot 0.33\ CH_2Cl_2$ and $[Ni(psq)_2] \cdot 1.5$ THF.

$Fe(psq)_2 \cdot 0.33CH_2Cl_2$		$[Ni(psq)_2] \cdot 1.5THF$	
Atom	D/Å	Atom	D/Å
Fe1-N1	2.17(1)	Ni1-N1	2.081(2)
Fe1-N2	2.10(1)	Ni1-N2	2.020(2)
Fe1-N3	2.19(1)	Ni1-N3	2.094(2)
Fe1-N4	2.161(9)	Ni1-N4	2.072(3)
Fe1-N5	2.085(8)	Ni1-N5	2.024(3)
Fe1-N6	2.161(8)	Ni1-N6	2.107(2)

Table S3. *Cis* bond angles measured for $Fe(psq)_2 \cdot 0.33CH_2Cl_2$ and $[Ni(psq)_2] \cdot 1.5THF$

$[Fe(psq)_2] \cdot 0.33CH_2Cl_2$		$[Ni(psq)_2] \cdot 1.5THF$	
atom	θ / °	atom	θ / °
N1-Fe1-N2	76.4(4)	N1-Ni1-N2	80.18(9)
N1-Fe1-N4	97.9(4)	N1-Ni1-N4	92.1(1)
N1-Fe1-N5	98.2(4)	N1-Ni1-N5	97.58(9)
N1-Fe1-N6	93.6(3)	N1-Ni1-N6	93.21(9)
N2-Fe1-N3	79.0(4)	N2-Ni1-N3	83.15(4)
N2-Fe1-N4	95.5(4)	N2-Ni1-N4	96.4(1)
N2-Fe1-N5	108.6(4)	N2-Ni1-N5	101.00(9)

Table S4. Trans bond angles measured for $Fe(psq)_2 \cdot 0.33CH_2Cl_2$, $[Ni(psq)_2] \cdot 1.5THF$.

$[Fe(psq)_2] \cdot 0.33CH_2Cl_2$		$[Ni(psq)_2] \cdot 1.5THF$	
atom	θ / °	atom	θ / °
N1-Fe1-N3	154.54(3)	N1-Ni1-N3	163.12(9)
N4-Fe1-N6	155.19(4)	N4-Ni1-N6	162.4(1)

Table S5. Octahedral distortion parameters (D, ζ, ∑ and θ) calculated using Octadist.

psq						
Compound	D[a] / Å	ζ[b] / Å	∑[c] / °	θ[d] / °	Bite angle / °	$\angle_{py\text{-}qu}$[e] / °
$Fe(psq)_2$	2.15	0.20	111.8	412.7	155	12.6
$[Co(psq)_2]^+$	1.939	0.134	45.52	162.3	170	8.02
$Co(psq)_2$	2.117	0.209	87.19	303.9	160	7.24
$Ni(psq)_2$	2.066	0.177	78.50	258.1	163	7.39
$Zn(psq)_2$	2.163	0.189	109.1	393.8	156	9.51
**terpy**						
Compound	D[a] / Å	ζ[b] / Å	∑[c] / °	θ[d] / °	Bite angle / °	$\angle_{py\text{-}py}$ / °
$[Fe(terpy)_2]^{2+}$	1.942	0.237	75.79	258.4	162	n/a
$[Co(terpy)_2]^{3+}$	1.918	0.233	64.25	210.2	165	n/a
$[Co(terpy)_2]^{2+}$	2.133	0.312	125.8	407.2	151	n/a
$[Ni(terpy)_2]^{2+}$	2.078	0.311	108.5	354.9	155	n/a
$[Zn(terpy)_2]^{2+}$	2.155	0.254	133.3	415.6	150	n/a

[a]Mean M-donor bond length, [b]the sum of the deviation from the mean bond length, [c]the sum of the deviations from 90° of the *cis* angles, [d]the sum of the deviation from 60° of the 24 possible torsional angles between the ligand atoms on the opposite triangular faces of the octahedron. In other words, the degree of twist towards a trigonal prismatic geometry. [e] Angle between the planes of the quinoline and pyridine rings.

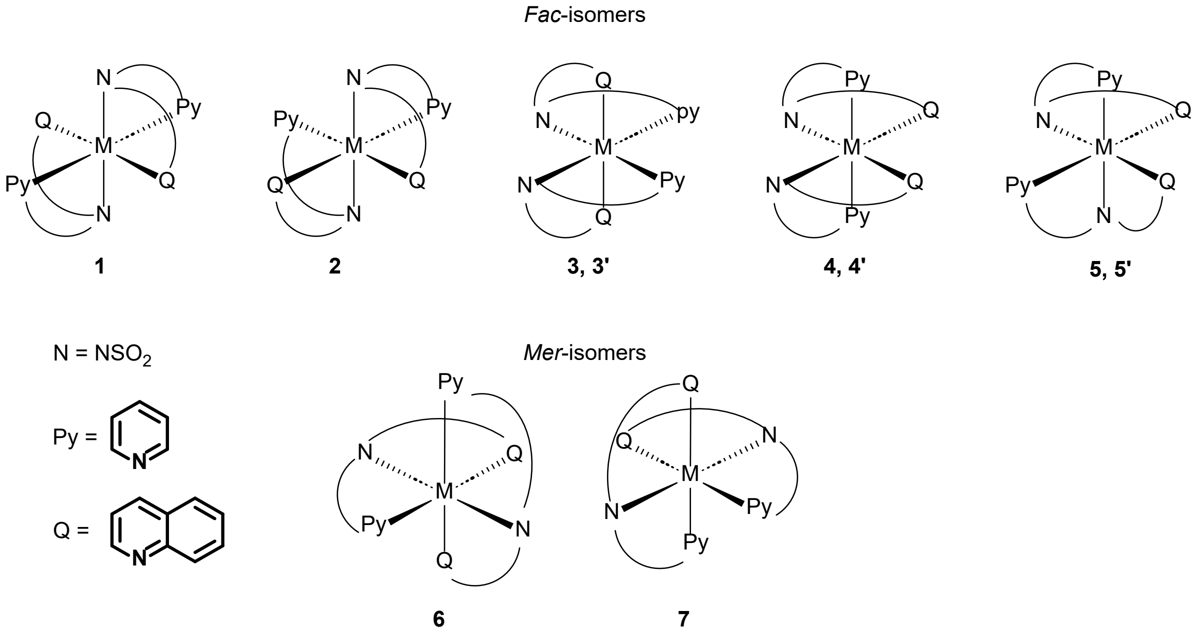

Scheme S2. Facial and meridional isomers of $M(psq)_2$ complexes. Three of the *fac* isomers (3, 4 and 5) have enantiomers (3', 4' and 5'). The *mer* isomers are enantiomers which are disordered in the crystal structures of the Co(II) and Co(III) complexes (CCDC ref codes SEFSUV, SEFSOP).

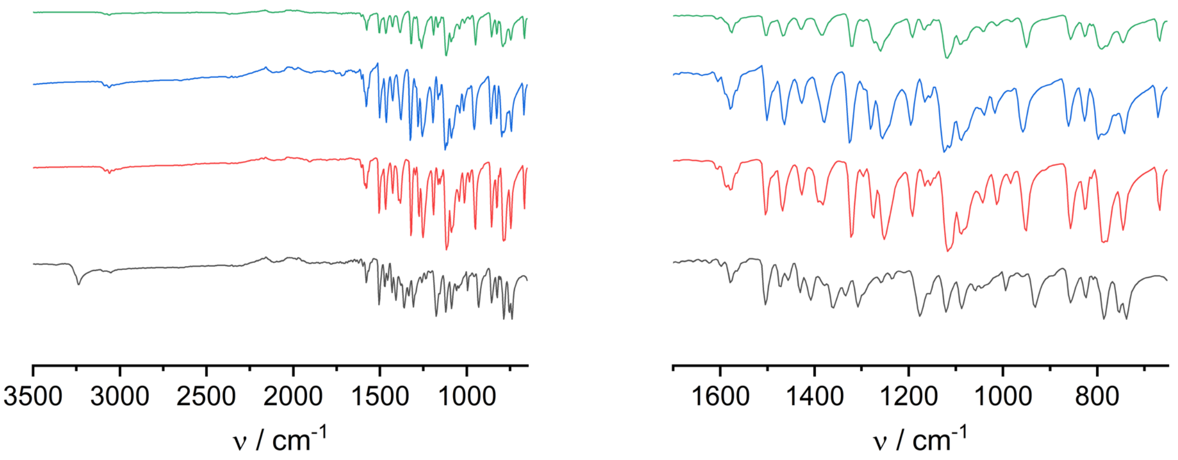

Figure S1. IR-spectra of $Ni(psq)_2$ (green), $Mn(psq)_2$ (blue), $Fe(psq)_2$ (red) and Hpsq(black).

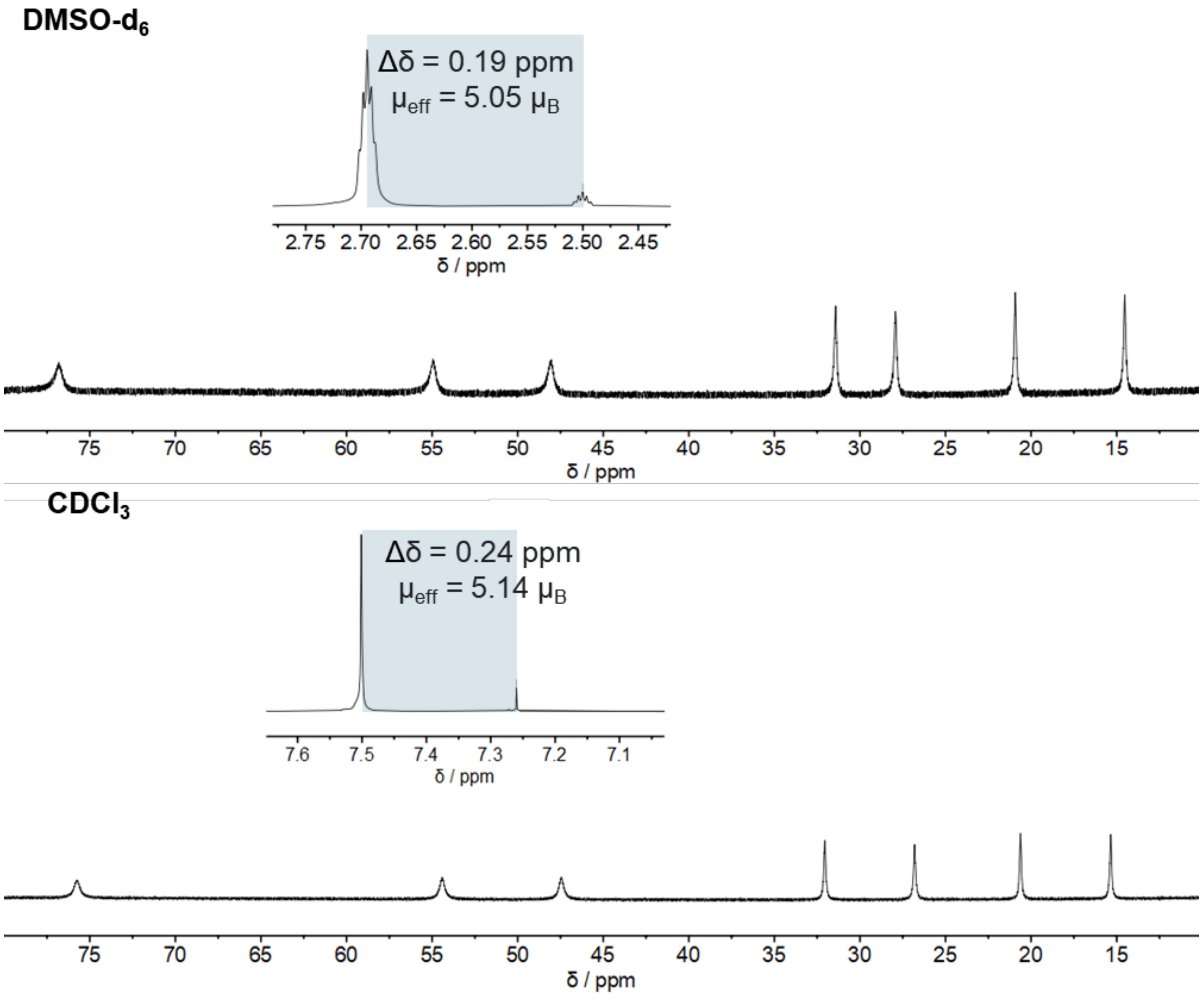

Figure S2. ^{1}H-NMR (80-10 ppm) of $Fe(psq)_2$ in $CDCl_3$ and DMSO-d_6.

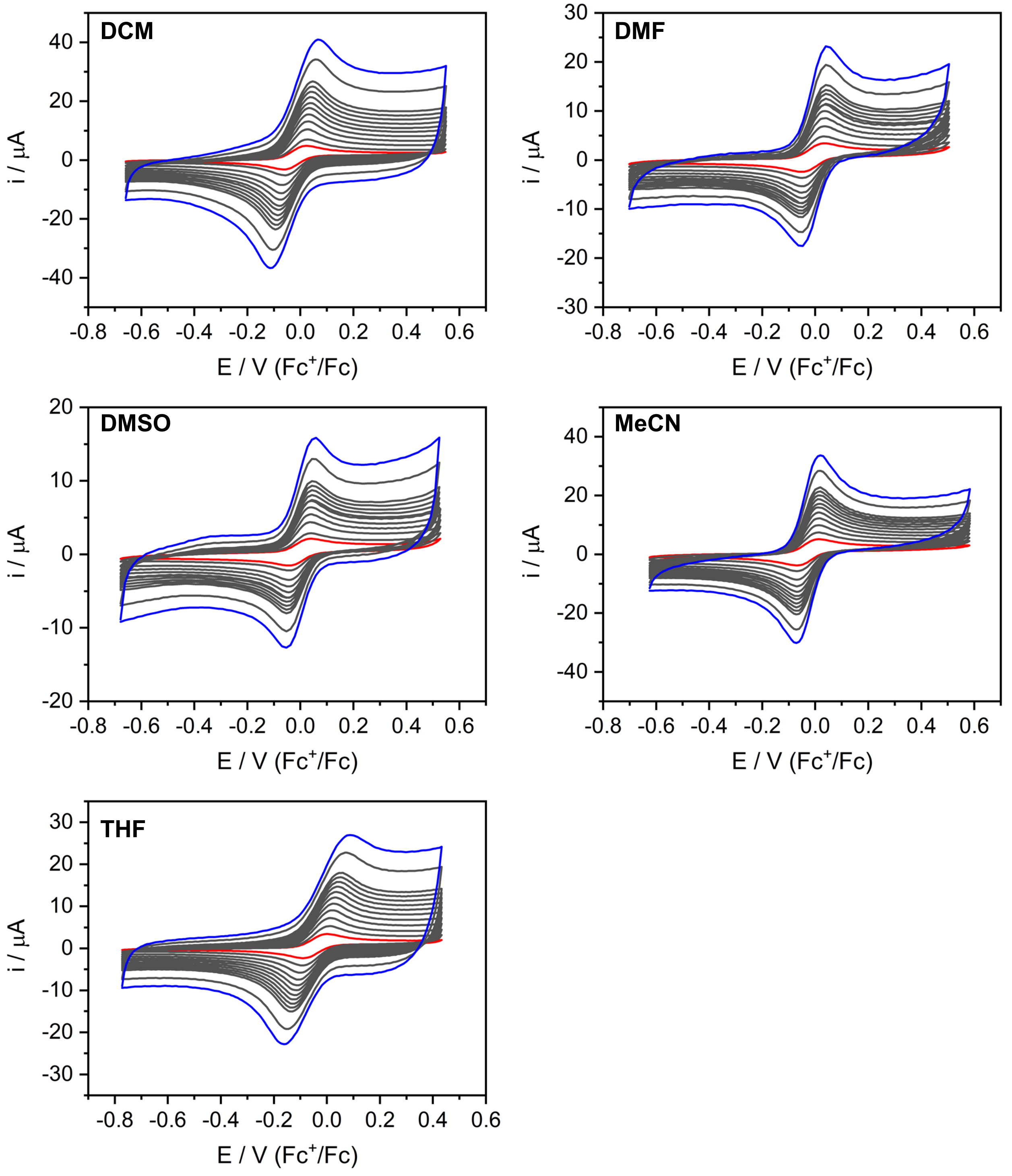

Figure S3. Scan rate dependent CV of $Fe(psq)_2$ (5 mM) in DCM, DMF, DMSO, MeCN and THF (0.1 M $TBAPF_6$) using a glassy carbon electrode.

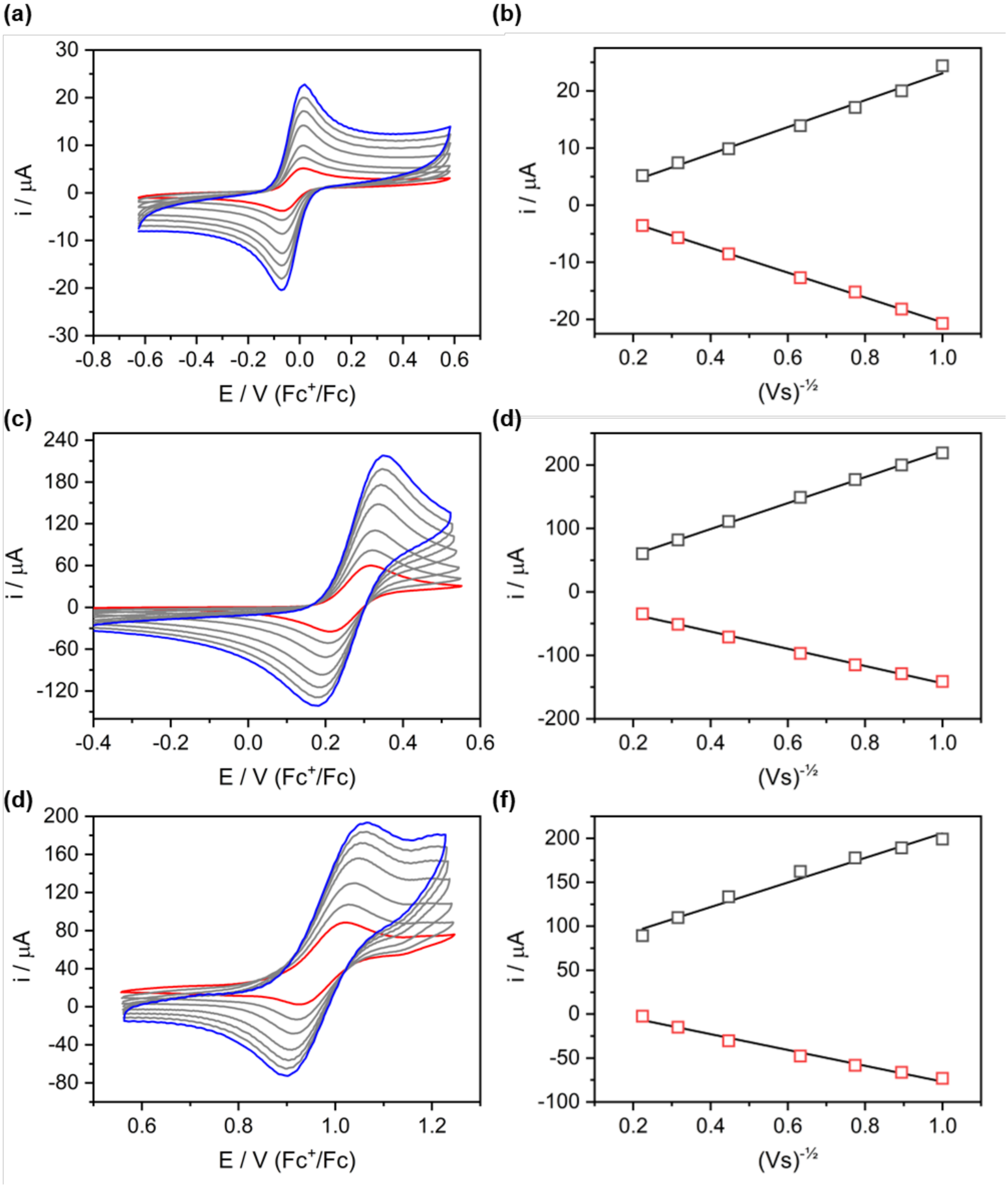

Figure S4. Cyclic voltammograms in MeCN (0.1 M $TBAPF_6$) of $Fe(psq)_2$ (a), $Mn(psq)_2$ (c,d) and the corresponding Randles-Sevik plots for $Fe(psq)_2$ (b) and $Mn(psq)_2$ (d,f).

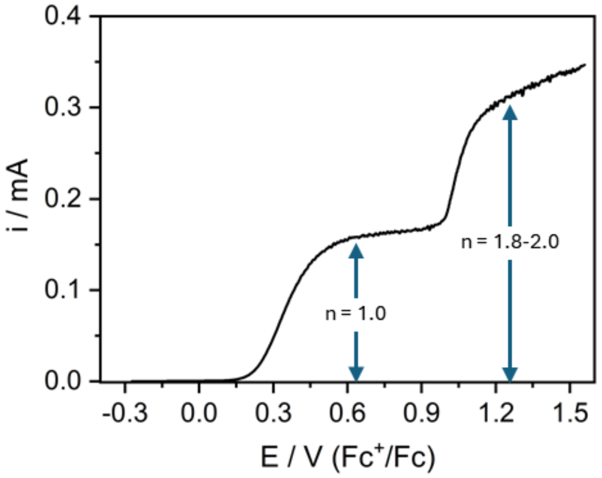

Figure S5 Linear Sweep Voltammetry-Rotating Disk Electrode (LSV-RDE) measurement for Mn(psq)$_2$ in MeCN.

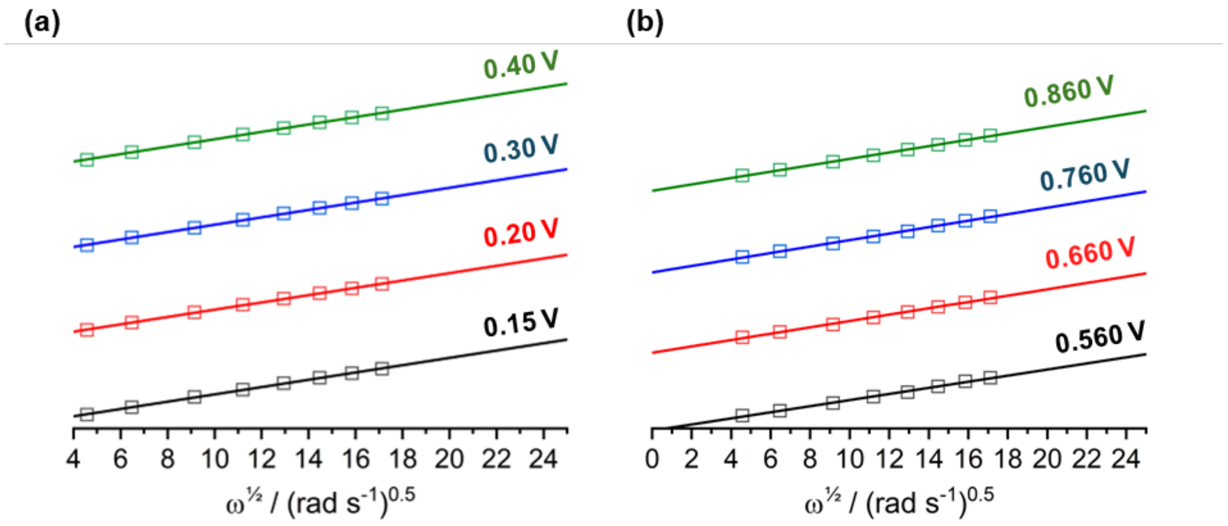

Figure S6. Levich plot of Fe(psq)$_2$ and Mn(psq)$_2$ in MeCN (0.1 M TBAPF$_6$) at applied potentials V.

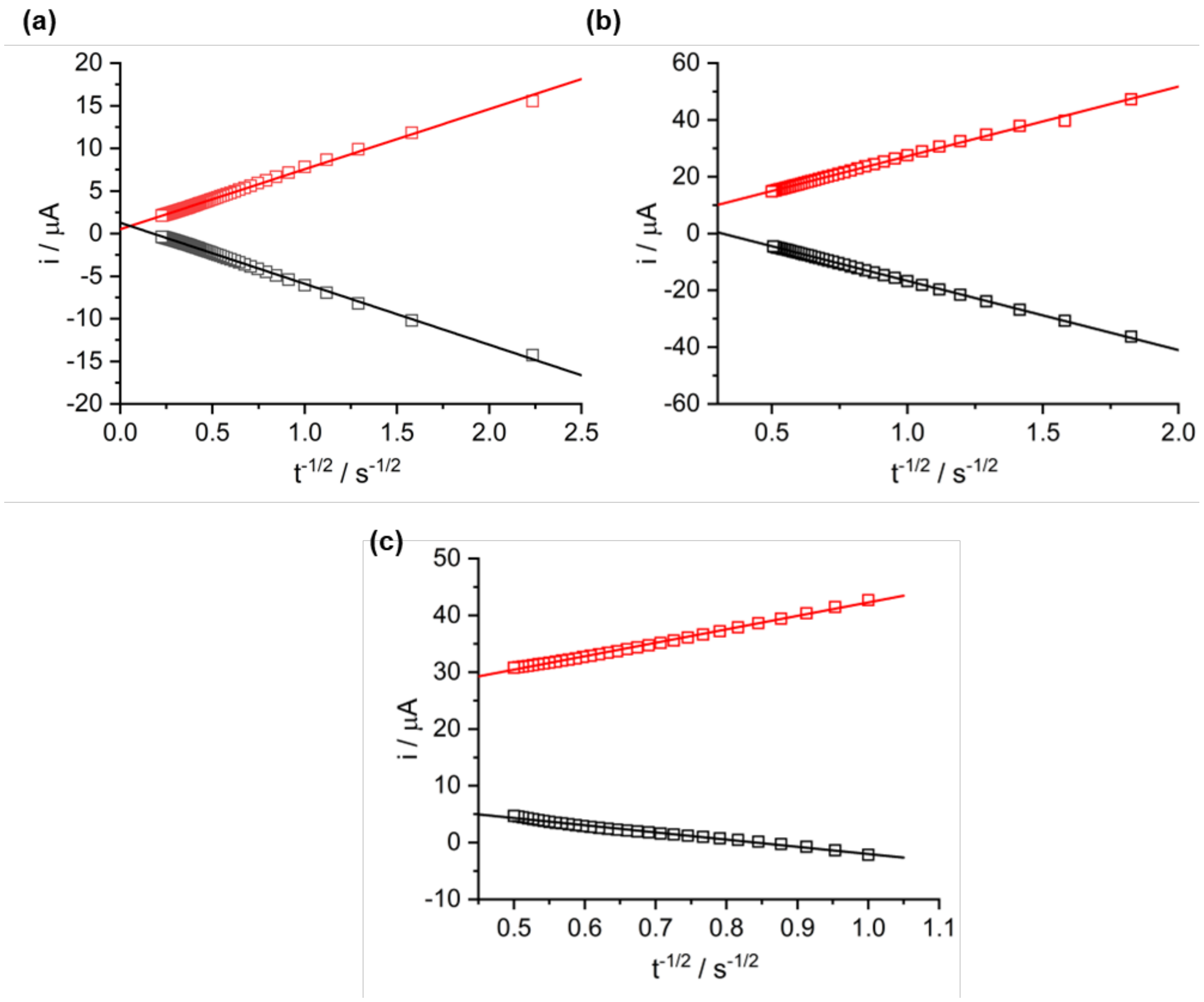

Figure S7. Cotrell plots for (a) $Fe(psq)_2$, (b) Mn^{III}/Mn^{II} and (c) Mn^{IV}/Mn^{II} for $Mn(psq)_2$

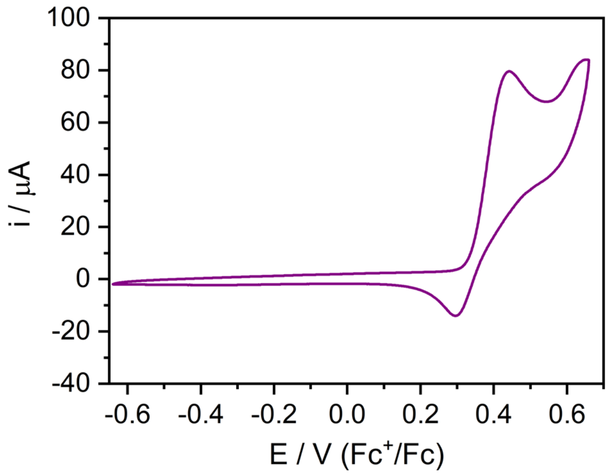

Figure S8. Cyclic voltammogram of $Ni(psq)_2$ (5 mM) in MeCN (0.1 M, $TBAPF_6$) at a glassy carbon electrode with 100 mV/s.

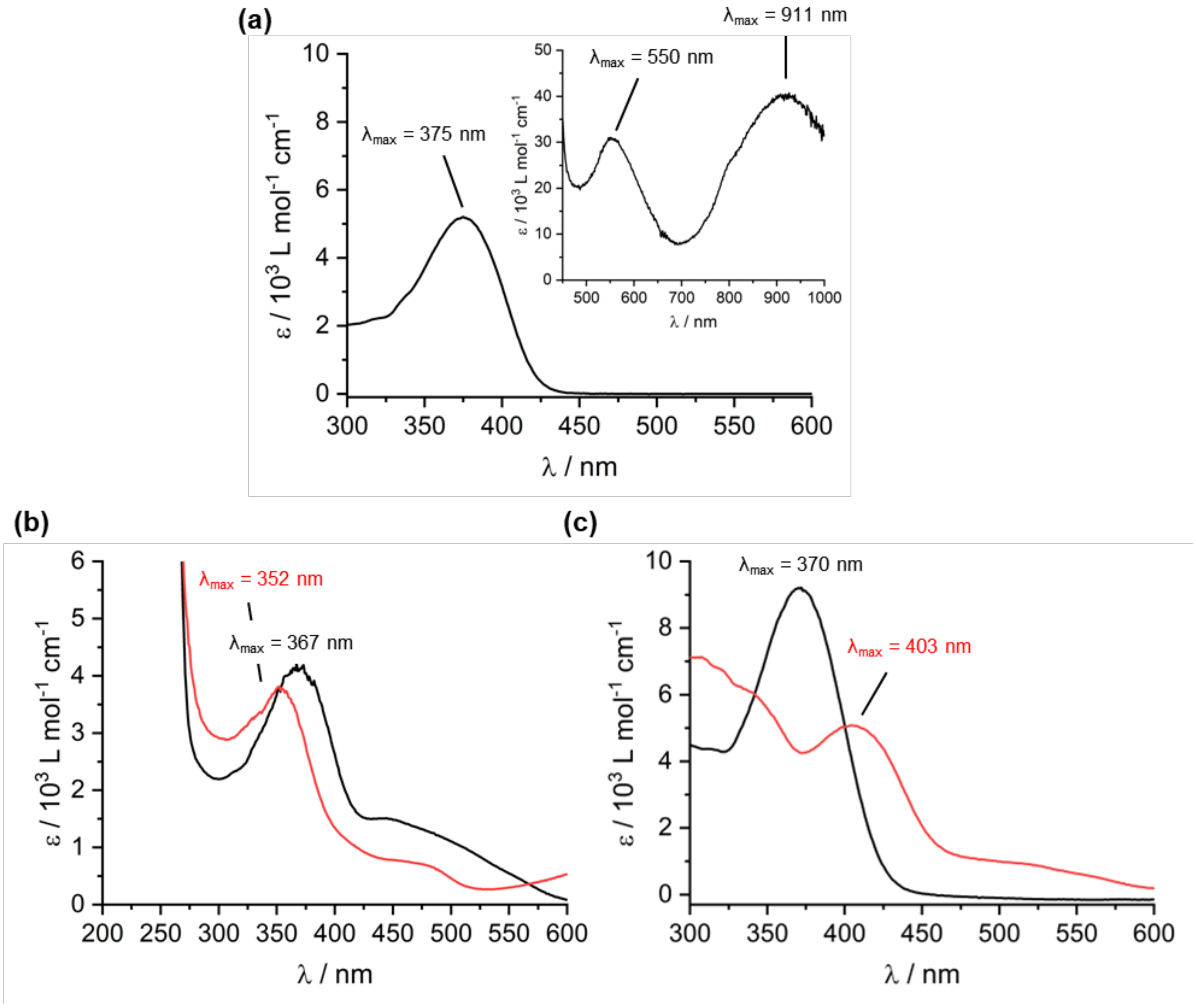

Figure S9. UV/vis absorption spectra of (a) $Ni(psq)_2$, (b) $Fe(psq)_2$ in the presence (red) and absence (black) of cerium ammonium nitrate (CAN) and (c) $Mn(psq)_2$ in the presence (red) and absence (black) of CAN.
